# Postoperative healing and recurrence in osteonecrosis of the jaw: influence of risk factors and surgical approach

**DOI:** 10.1007/s00784-026-06851-6

**Published:** 2026-03-28

**Authors:** Jonathan Mohr, Katharina Pippich, Claire Cherdron, Jannik Ketschau, Pia Erben, Helena Kram, Nils Krautkremer, Herbert Deppe, Klaus-Dietrich Wolff, Lucas M. Ritschl

**Affiliations:** Department of Oral and Maxillofacial Surgery, School of Medicine and Health, TUM University Hospital Klinikum Rechts der Isar, Technical University of Munich, Ismaninger Strasse 22, D-81675 Munich, Germany

**Keywords:** Osteonecrosis of the jaw, Antiresorptive-related osteonecrosis of the jaw (ARONJ), Osteoradionecrosis (ORN), Therapeutic management, Risk factors, Wound healing disturbances

## Abstract

**Objectives:**

The aim of this study was to evaluate the outcomes of surgical and adjunctive treatments for osteonecrosis of the jaw (ONJ) and to analyze the influence of specific risk factors on postoperative wound healing and recurrence rates.

**Materials and methods:**

We retrospectively analyzed surgically treated ARONJ and ORN patients from 2012 to 2017. Demographics, comorbidities, etiology, treatments, and healing outcomes were analyzed using descriptive statistics and multivariate regression analyses.

**Results:**

*N* = 194 patients met inclusion criteria. The overall postoperative healing rate was 78.4%, with wound healing disturbances occurring in 44% of cases. Healing rates were comparable between entities, with 79.4% in ORN and 77.3% in ARONJ patients achieving complete mucosal healing. Neither classical risk factors (smoking, obesity, diabetes) nor treatment regimens predicted wound healing disturbances (WHD). Risk factors for the development of ONJ were not consistently predictive of postoperative complications.

**Conclusions:**

Thorough resection with tension-free closure is central to ARONJ and ORN treatment. Close postoperative monitoring – especially within the first 100 days – is crucial, given the high early recurrence rate.

**Clinical relevance:**

The findings underscore the importance of surgical intervention – particularly complete necrotic bone resection with tension-free wound closure – as the cornerstone of effective treatment for osteonecrosis of the jaw, irrespective of its origin (ARONJ or ORN). Traditional risk factors did not predict wound-healing complications. Within the limitations of this retrospective study, common adjunctive measures did not appear to influence healing outcomes. Given the high rate of early recurrence, vigilant postoperative follow-up—especially within the first 100 days—is essential for optimal patient management.

## Introduction

Osteonecrosis of the jaw (ONJ) is defined as the pathological death of bone tissue in the maxillofacial region, clinically presenting as exposed bone that persists without healing for more than eight weeks [007/100OL, #40214] [1]. Osteonecrosis of the jaw can arise as a complication following head and neck radiotherapy, referred to as osteoradionecrosis (ORN) [2, 3], or as a side effect of antiresorptive therapy using bisphosphonates or RANKL (Receptor activator of nuclear factor kappa B ligand) inhibitors such as denosumab, termed antiresorptive-related osteonecrosis of the jaw (ARONJ) [4]. While the broader term medication-related osteonecrosis of the jaw (MRONJ) is increasingly used internationally, we use the term ARONJ throughout this manuscript in accordance with the current German S3 guideline, as the present cohort exclusively included cases associated with antiresorptive agents.

Historically, necrosis of the jaw was already recognized in the 19th century, when industrial workers exposed to white phosphorus developed the so-called “phossy jaw” [5, 6]. The first clinical description of radiation-induced jaw necrosis was provided by Regaud in 1922, documenting ORN following radium treatment of head and neck tumors [2, 3]. The modern concept of ARONJ was introduced by Marx in 2003 [007/100OL, #40214] [7], reflecting the increasing relevance of antiresorptive therapies in oncology and osteoporosis management according to demographic development.

Epidemiological data reveal a wide range of prevalence rates, influenced by underlying diseases, treatment protocols, and patient-specific risk factors [8, 9]. In patients with osteoporosis receiving low-dose antiresorptive therapy, the prevalence of ARONJ ranges from 0 to 0.5% [10, 11], while oncological patients undergoing high-dose regimens display prevalence rates of 1–21%, depending on additional risk factors such as concomitant chemotherapy or corticosteroid use [12–15]. Denosumab, although mechanistically different from bisphosphonates, has demonstrated similar ARONJ incidence rates in clinical trials [16, 17].

In ORN, the reported prevalence varies from 0 to 23% [18], with risk significantly increasing beyond cumulative radiation doses of 60 Gy (Gy) [19, 20]. Although modern radiotherapy techniques such as intensity-modulated radiotherapy (IMRT) have reduced the incidence of ORN, it remains a significant clinical challenge [21].

The pathogenesis of ONJ is considered multifactorial. In ARONJ, excessive suppression of bone turnover [22], microbial biofilm infections [23], and impaired immune responses [24] are regarded as major contributors. Osteoradionecrosis is primarily linked to radiation-induced fibrosis, vascular damage, and impaired bone remodeling [25]. In both entities, the mandible is predominantly affected due to its lower vascularization and higher mineral density, leading to greater susceptibility to mechanical stress and radiation injury [25]. Epidemiological studies report that approximately two-thirds of cases involve the mandible, while only a minority affect both jaws simultaneously [26]. Various patient-related risk factors further modulate the likelihood of ONJ development, including diabetes mellitus, smoking, advanced age, obesity, and concomitant immunosuppressive therapies [27–29]. Importantly, many of these risk factors negatively impact wound healing, posing additional challenges for surgical management.

Clinically, ONJ leads to significant morbidity, including chronic pain, impaired mastication and speech, facial disfigurement, and a considerable reduction in quality of life [4, 30]. The number of affected individuals continues to increase [4, 30] because of the increasing use of antiresorptive therapies and advances in oncologic treatment prolonging patient survival. Consequently, ONJ has become an increasingly important concern in oral and maxillofacial surgery. However, despite improvements in diagnostic and therapeutic strategies, the management of ONJ remains complex and controversial. While conservative approaches can be successful in early stages, surgical intervention is often required in advanced disease, and may be advantageous in early stages. The optimal timing, extent of resection, and adjunctive measures to enhance healing remain subjects of ongoing debate [8, 9].

Against this background, the present study aimed to retrospectively evaluate the therapeutic outcomes of surgical and adjunctive interventions for ONJ and to analyze the influence of patient- and disease-specific risk factors on postoperative wound healing and recurrence rates.

## Materials and methods

### Ethical statement and study collective

All clinical investigations were conducted according to the principles expressed in the Declaration of Helsinki. This retrospective study was approved by the institutional ethics committee of the Technische Universität München, Klinikum rechts der Isar (Approval Number: 309/19 S-SR).

This study included only patients who were surgically treated because of ARONJ or ORN between January 2012 and December 2017 at the Department of Oral and Maxillofacial Surgery, TUM University Hospital Klinikum Rechts der Isar, School of Medicine and Health, Technical University of Munich, Germany. All patients underwent surgical therapy with the aim of complete resection of necrotic bone and coverage with vital soft tissue. The standard approach involved marginal bone resections combined with tension-free primary wound closure whenever possible. In patients with multiple surgical interventions beyond 2017, the most recent hospital admission was selected to ensure evaluation of the latest therapeutic outcomes. Patient inclusion was based on the ICD (International classification of Diseases) code K10.28, representing “other specified inflammatory conditions of the jaws,” including ARONJ and ORN.

Diagnosis of ARONJ was established according to the criteria outlined by the German national S3 guideline [9], while the diagnosis of ORN was based on the German S2k guideline on infected osteoradionecrosis of the jaws [8]. In all cases, histopathological confirmation of necrosis was mandatory for inclusion. Imaging modalities and clinical examination supported the diagnosis. Patients with insufficient clinical documentation or who did not meet the diagnostic criteria for ARONJ or ORN were excluded. Patients treated exclusively with conservative therapy and cases without histopathological confirmation of necrosis were also excluded. No minimum postoperative follow-up duration was required for inclusion due to the retrospective design of the study. Only cases associated with antiresorptive medication were included; patients with osteonecrosis related to other drug classes, such as antiangiogenic agents, were excluded.

### Data collection and documentation

Patient data were collected retrospectively from multiple sources, including the hospital’s electronic medical record system, paper-based patient files, and records from the Department of Oral and Maxillofacial Surgery outpatient clinic. Data extraction focused on clinical, demographic, and treatment-related variables relevant to the diagnosis, therapy, and postoperative course of osteonecrosis of the jaw. Collected variables included patient demographics (age at surgery, gender, height, weight, body mass index, ASA (American Society of Anesthesiology) classification for physical status), lifestyle factors (e.g., smoking status), and comorbidities (e.g., diabetes mellitus, anticoagulant therapy, history of chemotherapy). Disease-specific parameters such as localization (subdivided into sextants), extent, and entity of necrosis (ARONJ, ORN) were also documented.

Therapy-related data included the type and duration of preoperative and postoperative antibiotic regimens, surgical techniques for wound closure, and the use of fluorescence-guided resection methods. In this study, “preoperative antibiotic therapy” referred to oral antibiotic treatment that had already been initiated prior to hospital admission, typically prescribed in the outpatient setting due to clinical signs of infection. Patients admitted for surgical treatment routinely received perioperative intravenous antibiotic therapy during their inpatient stay. Fluorescence-guided resection was applied in selected cases to assist intraoperative delineation between necrotic and viable bone. The decision to use fluorescence imaging was made on a case-by-case basis by the operating surgeon and was not based on a predefined study protocol.

For ARONJ patients, risk group classification was performed according to the German S3 guideline on antiresorptive-associated osteonecrosis of the jaw [9]. Surgical defect reconstruction primarily employed local flap techniques – including mucoperiosteal advancement flaps, buccal fat pad flaps, and mylohyoid muscle flaps. Free flap reconstruction was performed in cases involving extensive defects or when previous surgery or radiotherapy had led to a lack of sufficient soft tissue to allow for primary wound closure. In cases of extensive necrosis or major soft tissue defects and to protect the surgical site, enteral nutritional support was provided via nasogastric or percutaneous endoscopic gastrostomy (PEG) tubes. Postoperative management strategies, including extended antibiotic therapy, were also extracted.

Furthermore, postoperative outcomes were systematically recorded. Wound healing disturbance (WHD) was defined as postoperative wound dehiscence with exposed bone. Complete healing was defined as complete mucosal coverage of the surgical site without exposed bone during follow-up. Disease recurrence was defined as secondary bone exposure after initial mucosal healing.

### Data preparation

All collected data were entered into a preformatted Microsoft Excel spreadsheet and pseudonymized in accordance with the General Data Protection Regulation. No patient-identifiable information was used during data processing or statistical analysis. To prepare the dataset for statistical analysis, temporal intervals were calculated based on relevant clinical events, including the time from the initiation of antiresorptive therapy or radiotherapy to the onset of osteonecrosis, the time from previous surgeries to the reference surgery, and the duration of postoperative follow-up. These intervals were computed in days.

Categorical variables such as the type and localization of necrosis, risk group classification according to antiresorptive therapy guidelines [9], and presence of postoperative complications were systematically coded. Numerical variables were recorded in their continuous form when appropriate (e.g., age, body mass index, radiation dose). All data were cross-checked for plausibility and completeness prior to statistical evaluation.

### Statistical analysis

Statistical analysis was performed using IBM SPSS Statistics for Windows, version 28.0 (IBM Corp., Armonk, NY, USA) and R Version 4.4.2. Categorical variables were expressed as absolute and relative frequencies, while continuous variables were reported as means ± standard deviation, medians and interquartile range (IQR), or minimums and maximums.

Comparisons between groups were conducted using the chi-squared test or Fisher’s exact test for categorical variables, and either the independent t-test or the Wilcoxon rank-sum test (Mann–Whitney U test) for continuous variables, as appropriate. Logistic regression analyses were performed to identify independent risk factors associated with postoperative wound healing disturbances. All statistical tests were two-sided.

A *p*-value of less than 0.05 was considered statistically significant. All analyses were exploratory in nature, and no adjustments for multiple comparisons were made.

## Results

### Patient data

A total of 247 cases were initially identified that fulfilled the inclusion criteria. After adjusting for multiple admissions of the same patients and applying exclusion criteria – such as absence of histological confirmation, purely conservative management, or mismatch between clinical and histological diagnosis – a final study cohort of patient cases was established.

Two cases involved the same patient who presented with necrosis at different anatomical sites and at different time points; thus, they were counted separately.

A total of 194 patients were included in the final analysis. The mean age at the time of surgery was 67 years (44–95 years), with a standard deviation of 11 years. The majority of patients were male (59%, *n* = 114). Regarding the etiology of ONJ, 97 cases (50%) were classified as ARONJ and 97 cases (50%) as ORN. The mandible was affected in 79% of cases and bilateral lesions occurred in 4.1% of patients.

### Therapy and surgical procedures

Preoperative antibiotic therapy, referring to outpatient oral antibiotic treatment initiated before admission, was administered in 41% of patients and continued postoperatively, typically for a minimum of 1 to a maximum of 22 days depending on clinical progress.

Fluorescence-guided resection techniques were applied in selected cases on a case-by-case basis according to the surgeon’s intraoperative assessment. Intraoperative assessment of bone viability was based on fluorescence properties observed under specific light sources, without the routine use of photosensitizing agents. In total, 19 patients (9.8%) underwent fluorescence-guided surgery, the majority of whom (*n* = 17) were diagnosed with ARONJ. Consistent with current ARONJ guidelines, this technique was used as an optional adjunct. However, no significant advantage was observed in terms of postoperative wound healing outcomes in this cohort.

Regarding surgical wound closure, Rehrmann flaps or other local tissue advancement techniques were used in 54% of cases (*n* = 104), representing the most frequent approach. Free flap reconstruction was the second most common method, applied in 27% of patients (*n* = 52), followed by two-layer closures (18%; *n* = 34) and nasolabial flaps (2.1%; *n* = 4). Notably, patients with ORN underwent free flap procedures more often (44%), whereas those with ARONJ were more frequently treated with local flap techniques (67%). These differences were statistically significant (*p* < 0.001).

Postoperative enteral nutritional support was required in 84% of patients (*n* = 162). Among these, 134 patients received nasogastric feeding tubes, whereas PEG tubes – most of which had been placed due to the underlying disease – were present in 28 patients.

### Postoperative outcomes

Ultimately, complete mucosal healing was achieved in 78.4% of all cases (*n* = 152) during a median postoperative follow-up period of 37 days (IQR 12–125 days).

Temporary WHD during the early postoperative phase occurred in 44% of patients (*n* = 84) within a median of 13 days (IQR 10–29), typically coinciding with the first scheduled postoperative wound inspection. In most cases, these WHDs were minor and resolved with conservative management, leading to secondary mucosal closure in approximately 51.2% of affected patients.

In a subset of patients, however, secondary surgical revision (*n* = 16) was required to achieve stable mucosal coverage, typically consisting of repeat surgical treatment with renewed debridement and revision of the soft tissue closure. Overall healing rates were comparable between the two disease entities, with 79.4% in ORN and 77.3% in ARONJ patients achieving complete mucosal healing (Table 1). To account for the differing pathophysiology and causative agents of ARONJ and ORN, we performed a subgroup analysis comparing local versus free flap reconstruction within each disease entity (Table 2). This analysis revealed no statistically significant difference in wound healing outcomes between reconstruction types in either group.

Dehiscence with exposed bone was observed in 18 patients with ORN (18.6%) and in 19 patients with ARONJ (19.6%). The mean time to occurrence was 4.5 months following initial surgical treatment in the ORN group.

**Table 1:** General overview of clinical results in comparison between ARONJ and ORN

Variable	ARONJ	ORN	Total	*p*-value
Number of cases	97	97	194	
Sex Female Male	55 42	25 72	80 114	< 0.001^*^
Median age (IQR)	72 (65, 78)	63 (55, 69)	67 (60, 75)	< 0.001^^^
Mean age (SD)	71.1 (± 10.2)	63.2 (± 9.9)	67.0 (± 11)	< 0.001^^^
Localization of necrosis Maxilla Mandible Both	28 63 6	4 91 2	32 154 8	< 0.001^+^
Surgical wound closure Rehrmann flap/ local tissue advancement technique Two-layer closure Nasolabial flap Free flap	65 21 2 9	39 13 2 43	104 34 4 52	< 0.001^+^
Use of fluorescence-guided resection techniques	17	2	19	< 0.001^*^
Preoperative antibiotic treatment	45	34	79	0.095^*^
Duration of preoperative antibiotic treatment; median (IQR), days	2 (1, 3)	2 (1, 4)	2 (1, 3)	0.8^§^
Postoperative enteral nutritional support Nasogastric PEG	77 -	57 28	134 28	< 0.001^*^
Occurrence of wound healing disturbances (WHD)	41	44	84	0.7^*^
Interval operation – WHD; median (IQR)	13 (10, 30)	13 (10, 22)	13 (10, 29)	0.5^§^
Treatment of WHD Conservative Surgical (outpatient)	35 6	34 10	69 16	0.3^*^
Occurrence of exposed bone	19	18	37	0.6^*^
Interval operation – exposed bone; median (IQR)	30 (11, 57)	64 (15, 230)	38 (15, 101)	0.086^§^
Healing of WHD	19	24	43	0.5*
Interval occurrence WHD – healing; median (IQR)	35 (12, 88)	52 (15, 245)	39 (14, 101)	0.2^§^
Final healing rate	75	77	152	0.9^*^
Interval operation – last follow-up; median (IQR)	22 (11, 64)	59 (17, 213)	37 (12, 125)	< 0.001^§^

*ARONJ* antiresorptive-related osteonecrosis of the jaw, *ORN* osteoradionecrosis, *PEG* percutaneous endoscopic gastrostomy, *WHD* wound healing disturbance

* Chi-squared test; ^+^ Fisher’s exact test; ^ t-test; ^§^ Wilcoxon rank sum/Mann–Whitney U test

## Risk factor analysis

Multiple patient-specific and disease-specific factors were analyzed for their potential association with impaired postoperative wound healing and disease recurrence.

Univariate analysis for WHD in the ORN group showed no significant associations with any of the investigated variables. A trend toward increased risk was observed for the intake of antiplatelet drugs (OR 2.22, 95% CI 0.93–5.39).

In contrast, for WHD in ARONJ patients, male sex (*p* = 0.031), age (*p* = 0.048), and preoperative antibiotic treatment (*p* = 0.018) were identified as significant risk factors. Further trends were noted for the ARONJ high-risk group (OR 2.45, 95% CI 0.90–7.49) and the use of fluorescence-guided resection (OR 0.36, 95% CI 0.09–1.11). Multivariate analysis did not confirm any independent risk factors.

Risk factors for ONJ such as diabetes mellitus (*p* = 0.314; *p* = 0.495), Body mass index (BMI) (*p* = 0.241; *p* = 0.585), and smoking (*p* = 0.983; *p* = 0.883) did not show a statistically significant association with impaired postoperative healing in this cohort in either group (Tables 2 and 3).

**Table 2:** Overall risk factor analysis for the development of a wound healing disturbance

Variable	*p*-value	95% CI (coefficient)	OR (95% CI)
Age	0.137	-0.006–0.047	
Sex (Male)	0.156	-0.158–1.010	1.53 (0.85–2.75)
BMI	0.311	-0.030–0.093	
ASA status > 2	0.922	-0.614–0.553	
Smoking	0.992	-0.600–0.590	
Diabetes mellitus	0.257	-0.350–1.330	
Intake of antiplatelet drugs	0.235	-0.245–0.996	
Intake of anticoagulation drugs	0.235	-0.245–0.996	
Number of previous operations in the area	0.235	-0.079–0.330	
Entity (reference ARONJ)	0.724	-0.467–0.674	
ARONJ risk group (high)	0.094	-0.111–2.010	2.45 (0.89–7.49)
Drug holiday	0.177	-1.760–0.308	
Preoperative antibiotic treatment	0.083	-0.065–1.100	1.67 (0.94–3.01)
Postoperative enteral nutritional support	0.537	-0.529–1.060	
Type of Surgical wound closure (reference: Rehrmann flap/local tissue advancement technique)	Reference		
Two-layer closure Nasolabial flap	0.375	-1.13 − 0 0.41	
Free flap	0.991	-0.682–0.669	
Use of fluorescence-guided resection techniques	0.119	-2.01–0.16	0.43 (0.13–1.18)

*BMI* Body mass index, *ASA* American Society of Anesthesiologists (classification), *ARONJ* antiresorptive-related osteonecrosis of the jaw

**Table 3:** Risk factor analysis for the development of a wound healing disturbance in comparison between ARONJ and ORN

Variable	ORN			ARONJ		
	*p*-value	95% CI	OR (95% CI)	*p*-value	95% CI	OR (95% CI)
Age	0.666	-0.032–0.050		**0.048**	0.002–0.088	
Sex (Male)	0.708	-1.09–0.75	0.84 (0.36–2.11)	**0.031**	0.01–1.75	2.49 (1.10–5.78)
BMI	0.247	-0.039–0.162		0.585	-0.059–0.107	
ASA status > 2	0.623	-0.609–1.02		0.513	-1.13–0.557	
Smoking	0.983	-0.817–0.798		0.883	-1.06–0.886	
Diabetes mellitus	0.314	-0.636–2.11		0.495	-0.715–1.47	
Intake of antiplatelet drugs	0.074	-0.068–1.68	2.22 (0.93–5.39)	0.850	-1.01–0.809	
Intake of anticoagulation drugs	0.431	-3.95–1.17		0.847	-1.28–1.00	
Number of previous operations in the area	0.845	-0.252–0.305		0.141	-0.071–0.595	
Preoperative antibiotic treatment	0.925	-0.813–0.888	1.04 (0.44–2.43)	**0.018**	0.183–1.86	2.73 (1.20–6.41)
ARONJ risk group (high)				0.094	-0.111–2.01	2.45 (0.89–7.49)
Drug holiday				0.177	-1.76–0.308	
Postoperative enteral nutritional support	1.240	0.048–31.90	0.79 (0.23–2.71)	0.277	-0.443–1.72	1.81 (0.64–5.59)
Two-layer closure Nasolabial flap	0.149	-2.37–0.290		0.973	-0.961–0.974	
Free flap	0.921	-0.920–0.831		0.581	-2.04–1.01	
Use of fluorescence-guided resection techniques	0.881	-3.04–3.46	1.24 (0.05–31.90)	0.094	-2.36–0.10	0.358 (0.09–1.11)

*ARONJ* antiresorptive-related osteonecrosis of the jaw, *ORN* osteoradionecrosis

### Recurrence and long-term follow-up

During the postoperative follow-up period, WHD in patients with ORN requiring further outpatient surgical intervention was observed in 22.7% of patients (*n* = 10). Among patients with WHD (*n* = 84), 44% (*n* = 37) developed clinical recurrence, defined as recurrent exposed bone at the surgical site, with a median time to recurrence of 38 days (IQR 15–101). 75% of clinical recurrence occurred within the first 100 postoperative days; the latest recurrence was documented 528 days postoperatively.

Overall, long-term follow-up demonstrated sustained mucosal stability in the majority of successfully treated patients (78.4%), with no evidence of late disease progression in cases achieving complete initial healing.

## Discussion

This study retrospectively analyzed a large cohort of surgically treated patients presenting with osteonecrosis of the jaw, including cases of ARONJ and ORN. The overall healing rate of 78.4% observed in this study. In detail, healing rates were comparable between groups, with 79.4% in ORN and 77.3% in ARONJ patients achieving complete mucosal healing, which aligns with previously reported healing rates from 70% to 85% in the literature depending on disease entity and treatment strategy [8, 9]. The relatively low recurrence rate and the observation that most recurrences occurred within the first year postoperatively highlight the importance of close clinical monitoring during the early postoperative period. Long-term mucosal stability was achievable in the majority of patients with complete initial healing, supporting the efficacy of radical surgical resection combined with primary wound closure.

Consistent with previous findings, ARONJ patients demonstrated lower rates of WHD but slightly lower final healing rates compared with ORN patients [11, 12]. In contrast, ORN cases presented with a more variable and often extensive operative course, frequently necessitating major resections and reconstructive procedures due to radiation-induced tissue compromise. In addition, ORN patients in our cohort more frequently underwent multiple previous surgical interventions, likely reflecting the more complex local tissue conditions following radiotherapy, including fibrosis, reduced vascularization, and impaired wound healing capacity. Many of these previous procedures had been performed at referring institutions prior to presentation at our tertiary care center, which limited the availability of detailed information regarding the exact number and type of prior surgical interventions. Despite this more complex surgical history, final healing rates were comparable between ORN and ARONJ patients. Notably, approximately 44% of all cases in this cohort required microvascular free flap reconstruction, a remarkably high rate that may reflect the accumulation of advanced cases in our department and underscores the complexity of the treated population. These results emphasize the importance of careful risk stratification, early surgical intervention, and – particularly in high-risk patients – the implementation of preventive strategies to reduce the incidence and severity of osteonecrosis of the jaw.

### Risk factors

Interestingly, classic systemic risk factors such as diabetes mellitus, smoking, and obesity did not demonstrate a significant influence on healing outcomes in this cohort. This finding suggests that local factors, including prior surgical interventions and extent of necrosis, may have a greater impact on prognosis than systemic comorbidities. Histologic analysis of the size expansion of the ONJ was not possible in this retrospective study for two reasons. Firstly, for procedural reasons, since the standard procedure is a decortication wherein bone is removed locally without reference to each other (as it would be possible, for example, in the context of a segment or continuity resection). Secondly, measuring the extent of the resected bone *en bloc* would only be possible in the axial direction, which is not done by pathologists as standard.

### Role/Influence of surgical and adjunctive procedures

In this study, complete mucosal healing without recurrence of exposed bone was achieved in 78.4% of cases. The use of fluorescence-guided resection in selected cases may have contributed to improved intraoperative visualization of necrotic bone margins. However, this study did not specifically evaluate the clinical impact of fluorescence techniques on healing outcomes. Although fluorescence-guided surgery has become an established technique in osteonecrosis management, further high-level evidence from randomized controlled trials would be beneficial to consolidate its role.

Tension-free wound closure and the avoidance of dead space, in addition to proper intraoperative bleeding stimulation, play equally crucial roles in promoting mucosal healing. Various surgical techniques have been described in the literature with heterogeneous quality. In our cohort, a stepwise approach was applied, including periosteal-releasing incisions in the Rehrmann flap design and consistent subperiosteal preparation. This approach corresponds well with findings from a prospective randomized trial by Ristow et al., which showed significantly more exposed bone after epiperiosteal versus subperiosteal flap elevation during an eight-week follow-up period [31]. In mandibular defects, a mylohyoid flap was used to achieve a double-layer wound closure, primarily to eliminate dead space in pronounced concave defects. This approach is consistent with the findings of Mücke et al., who reported improved healing outcomes using this technique in the surgical management of ARONJ [32]. In extensive defects, free tissue transfer has proven to be a safe and long-term effective surgical treatment option, as also demonstrated in our study [33, 34]. In the future, the role and potential applications of platelet-rich fibrin (PRF) will be better understood. For example, Straub et al. (2022) have described that PRF, when applied to the decorticated bone surface, can serve as a reliable local bio-carrier for previously administered antibiotics [35].

The role of antibiotic prophylaxis – including the choice of agent, route of administration, and duration – cannot be conclusively determined from this study. Interestingly, preoperative antibiotic therapy was associated with wound healing disturbances in the ARONJ subgroup. In this study, this variable primarily reflected outpatient antibiotic treatment initiated prior to hospital admission in patients presenting with clinical signs of infection or inflammatory exacerbation. This likely represents confounding by indication, as these patients may have presented with more advanced or inflamed disease at baseline. No superiority of any specific regimen was observed. Current literature suggests that oral administration may be sufficient, for example, in the context of preventive dental extractions [36].

The strengths of this study include the relatively large sample size (*n* = 194), the uniform surgical approach, and the comprehensive follow-up. Limitations include its retrospective design, potential documentation biases, and the heterogeneity of patient comorbidities and adjunctive treatments, which may confound the interpretation of specific risk factors. In particular, the small number of fluorescence-guided resections (*n* = 19) precludes any robust statistical conclusions regarding its potential therapeutic benefit. Furthermore, formal staging of ARONJ and ORN was not included in the analysis, as the retrospective nature of the study and incomplete documentation in some cases did not allow for a reliable reconstruction of lesion extent.

Interestingly, neither the presence of a PEG nor the use of a nasogastric feeding tube showed any measurable impact on postoperative healing outcomes in this cohort. Complete mucosal healing was observed in patients regardless of the chosen route of nutrition, suggesting that oral intake versus enteral feeding did not significantly influence surgical success.

Ultimately, preventive measures should be prioritized to avoid the development of osteonecrosis in the first place. Preventive oral and maxillofacial treatment prior to the initiation of antiresorptive therapy, combined with three-monthly dental follow-ups, has been shown to significantly reduce the occurrence and risk of ARONJ. Therefore, this approach should be systematically implemented in clinical treatment algorithms [37].

## Conclusion

This study confirms that surgical intervention remains the cornerstone in the treatment of jaw necroses – both ARONJ and ORN – yielding favorable healing outcomes across etiologies when thorough decortication or resection combined with tension-free wound closure is achieved. Common systemic comorbidities such as smoking, diabetes mellitus, and obesity did not demonstrate a significant impact on healing success. Intensive perioperative management and early identification of high-risk patients are critical for improving long-term outcomes. Notably, 75% of recurrences occurred within the first 100 postoperative days, underscoring the importance of close follow-up during this period.

Future research should prioritize prospective, controlled studies to refine surgical strategies and validate adjunctive diagnostic approaches in the management of osteonecrosis of the jaw.

## Data Availability

No datasets were generated or analysed during the current study.

## Abbreviations

ARONJ: Antiresorptive-related osteonecrosis of the jaw
ASA: American Society of Anesthesiologists (classification)
BMI: Body mass index
CI: Confidence interval
Gy: Gray (unit)
ICD: International Classification of Diseases
IMRT: Intensity-modulated radiotherapy
IQR: Interquartile range
ONJ: Osteonecrosis of the jaw
OR: Odds ratio
ORN: Osteoradionecrosis
PEG: Percutaneous endoscopic gastrostomy
PRF: Platelet-rich fibrin
RANKL: Receptor activator of nuclear factor kappa-Β ligand
SD: Standard deviation
WHD: Wound healing disturbance
